# Death of Dr. F. E. Anstie

**Published:** 1874-11

**Authors:** 


*Death of Dr. F. E. Anstie.*

The following announcement from the *London Medical Record* for September 16th will bring sadness to the heart of many a practitioner of medicine in America, where Dr. Anstie was, through his able Journal, *The Practitioner*, known and honored as a high-toned gentleman and a conscientious and laborious scientific investigator. Our profession can ill afford to lose such men:

"With much regret we announce the death of Dr. F. E. Anstie, Physician to, and Lecturer at the Westminster Hospital. He died on Saturday last, after an illness of three days, brought on by exposure to sewer emanations while examining the sanitary defects of a school at Wadsworth early in the week. Dr. Anstie has made some valuable contributions to the medical literature on Alcohol, Neuralgia and other subjects. His death will be felt as a great loss to the profession." S. M. B.

